# Effect of Implant Angulation on Long-Term Outcomes of Screw-Retained Versus Cement-Retained Single Implant Crowns: A Retrospective Cohort Study

**DOI:** 10.7759/cureus.109036

**Published:** 2026-05-17

**Authors:** Tarannum Ajaz, Tayyeb S Khan, Rohit Patil, Jay Gohil, Lahari Priya Singareddy, Nitin Goyal

**Affiliations:** 1 Department of Prosthodontics, Purvanchal Institute of Dental Sciences, Gorakhpur, IND; 2 Department of Oral and Maxillofacial Surgery, Purvanchal Institute of Dental Sciences, Gorakhpur, IND; 3 Department of Prosthodontics, Jawahar Medical Foundation's Annasaheb Chudaman Patil Memorial Dental College, Dhule, IND; 4 Department of Prosthodontics, K. M. Shah Dental College and Hospital, Sumandeep Vidyapeeth Deemed to Be University, Vadodara, IND; 5 Department of Public Health, St. Francis College, New York, USA; 6 Department of Periodontology, Mahatma Gandhi Dental College and Hospital, Jaipur, IND

**Keywords:** cement-retained restorations, dental implants, implant angulation, prosthetic retention, screw-retained restorations

## Abstract

Introduction: Implant angulation and prosthetic retention are critical determinants of the long-term success of implant-supported restorations. However, there is limited evidence to evaluate the combined influence on prosthetic survival and complication rates. This study aimed to assess the effects of implant angulation on the outcomes of screw-retained and cement-retained implant-supported crowns.

Materials and methods: This retrospective cohort study was conducted using institutional electronic dental records. A total of 720 implant-supported single crowns with a minimum follow-up of five years were included. Cases were categorized based on the prosthetic retention type and stratified into angulation groups of 0-10°, 11-20°, and greater than 20°. Data on prosthetic survival, mechanical complications, and biological complications were also collected. Statistical analyses included independent samples t-test, chi-square test, Kaplan-Meier survival analysis, Cox proportional hazards regression, and receiver operating characteristic curve analysis.

Results: The mean age of participants was 52.4 ± 11.6 years. No significant differences were observed in baseline characteristics between the groups (p > 0.05). Prosthetic survival decreased with increasing angulation. The five‑year cumulative survival rates for screw‑retained crowns were 98.2% (0-10°), 94.5% (11-20°), and 88.7% (>20°); for cement‑retained crowns, survival rates were 96.8% (0-10°), 87.3% (11-20°), and 72.4% (>20°). Screw‑retained restorations demonstrated significantly better survival across all angulation categories (log‑rank p < 0.001). At angulations greater than 20°, cement-retained restorations showed higher rates of mechanical complications, including crown decementation and screw loosening, compared to screw-retained restorations (p < 0.001). Biological complications such as peri-implantitis were also higher in cement-retained restorations (p < 0.001). Regression analysis identified cement-retained restorations (hazard ratio, 1.42) and angulation greater than 20° (hazard ratio, 1.87) as significant predictors of complications.

Conclusion: Implant angulation had a significant impact on prosthetic outcomes, with higher angulations being associated with increased complication rates. Screw-retained restorations demonstrated a more favorable performance than cement-retained restorations, particularly at angulations greater than 20°. These findings highlight the importance of considering the implant angulation during treatment planning. A preference for screw-retained designs in highly angulated cases may improve long-term clinical success.

## Introduction

Implant-supported single crowns have become a predictable and widely accepted treatment modality for the rehabilitation of partially edentulous patients [1]. Among the key prosthetic considerations, the choice between screw-retained crowns and cement-retained crowns remains a topic of ongoing clinical debate [2]. Screw-retained restorations offer advantages, such as retrievability and elimination of excess cement-related complications, whereas cement-retained restorations provide improved esthetics and simplified occlusal morphology [2,3]. However, the long-term success of implant restorations is influenced by multiple biomechanical and biological factors, among which implant angulation plays a critical role [4].

Implant angulation, defined as the angular deviation of the implant’s long axis from the long axis of the alveolar ridge at the implant site, directly affects load distribution, prosthetic design feasibility, and complication risk [4]. With increasing angulation, screw access channels may become unfavorable, often leading clinicians to prefer cement-retained restorations. Although angled screw-channel technologies have expanded the applicability of screw-retained designs, evidence evaluating their performance across various angulations remains limited [5].

Recent research has emphasized the multifactorial nature of implant success and the importance of predictive analytics in identifying risk factors. Shahapur et al. [6] demonstrated the utility of decision tree regression models in identifying the predictors of implant failure, highlighting the role of clinical and anatomical variables in influencing outcomes. Similarly, advances in artificial intelligence have enabled the automated prediction of implant success using cone-beam computed tomographic (CBCT)-based imaging data, as shown by Taide et al. [7], reinforcing the importance of preoperative anatomical assessment in treatment planning. From a biological perspective, peri-implant tissue stability is a key determinant of long-term success. Studies reported that implant design factors significantly influence peri-implant bone loss, underscoring the interplay between prosthetic design and biological outcome [8]. Despite these advances, comprehensive studies evaluating the combined influence of implant angulation and prosthetic retention type on long-term survival and complication rates are lacking.

This retrospective cohort study aimed to assess the influence of implant angulation on prosthetic survival and complication rates in screw-retained and cement-retained implant-supported crowns. The objectives of the study were to (1) compare outcomes across different angulation categories (0-10°, 11-20°, and greater than 20°); (2) determine a critical angulation threshold associated with an increased risk of complications; and (3) evaluate the influence of clinical and demographic variables on treatment outcomes.

## Materials and methods

Study design

This study was designed as a retrospective cohort study conducted in accordance with the Strengthening the Reporting of Observational Studies in Epidemiology guidelines [9]. The unit of analysis was an individual, implant-supported single crown. Participants were categorized based on prosthetic retention type into screw-retained crowns and cement-retained crowns, and further stratified according to implant angulation into three categories: 0° to 10°, 11° to 20°, and greater than 20°. Each patient contributed only one implant crown; consequently, no patient contributed multiple implants, and clustering effects were not present.

Study setting and duration

This study was conducted at the Department of Prosthodontics at the Jawahar Medical Foundation's Annasaheb Chudaman Patil Memorial Dental College, Dhule, Maharashtra, India. The patient records from January 2012 to December 2019 were reviewed. Only cases with a minimum follow-up duration of five years after prosthesis delivery were included. Data extraction and analysis were performed between January 2024 and June 2024. Ethical approval was obtained from the institutional ethics committee prior to the data collection. As this was a retrospective study using anonymized patient records, a waiver for informed consent was granted. All data were de-identified prior to analysis. This study adhered to the principles of the Declaration of Helsinki.

Selection of records and cohort formation

**Figure 1:**
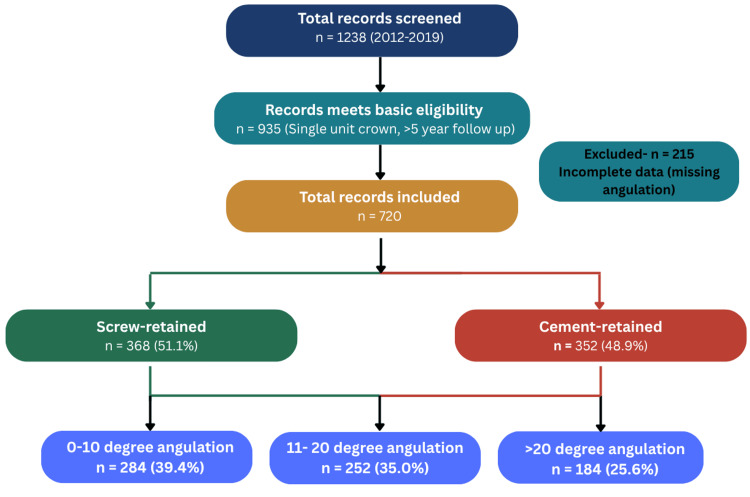
Flow diagram illustrating record screening, eligibility assessment, exclusions, and final allocation into screw-retained and cement-retained groups with angulation-based subgroups. Data are presented as n (%). Exclusion was based on incomplete data (missing angulation measurements). Angulation was categorized as 0-10°, 11-20°, and >20°.

Since this retrospective study utilized a census-based sampling approach, all available records of patients who received implant-supported single crowns during the study period were screened from the institutional electronic dental record database. Records were evaluated according to predefined inclusion and exclusion criteria. Patients with incomplete documentation, missing implant angulation data, insufficient follow-up, or those involving multi-unit prostheses were excluded. Following screening, 720 implant-supported single crowns were included in the final analysis, comprising both screw-retained and cement-retained restorations. The detailed selection process is illustrated in Figure 1.

Participant selection criteria

Adult patients aged ≥ 18 years who received a single implant-supported crown and had complete clinical and radiographic records were included. Implants were required to have documented angulation measurements and a minimum of five years of follow-up. Patients with systemic conditions affecting osseointegration, incomplete records, failed bone grafts, or insufficient follow-up visits were excluded from the study. Cases involving implant-supported fixed partial dentures or full-arch prostheses were excluded.

Data collection and variables

Data, including demographic details, implant characteristics, prosthetic details, and follow-up outcomes, were extracted from the institutional electronic dental records system. Screw-retained restorations were identified based on documentation of screw access channels and the absence of luting cement, whereas cement-retained restorations were identified based on the recorded use of dental cement in clinical and laboratory records. The primary outcome variables included prosthetic survival, prosthetic success, and occurrence of mechanical and biological complications.

Mechanical complications included: screw loosening - defined as clinically detectable loosening of the abutment screw for cement‑retained restorations or loosening of the prosthetic screw for screw‑retained restorations, confirmed by attempted retightening; screw fracture - fracture of either the abutment screw (cement‑retained) or prosthetic screw (screw‑retained); crown decementation - complete loss of retention of a cement‑retained crown; and abutment fracture - fracture of the implant abutment (applicable only to cement‑retained restorations, as screw‑retained restorations in this study used one‑piece screw‑retained crowns without separate abutments).

Biological complications included peri-implant mucositis and peri-implantitis in accordance with the 2017 World Workshop on the Classification of Periodontal and Peri‑Implant Diseases and Conditions.

Measurement methods

Implant angulation was measured using CBCT images with cross-sectional reconstruction aligned to the long axis of the alveolar ridge. Measurements were performed using Planmeca Romexis software (Planmeca Oy, Helsinki, Finland). Two calibrated examiners independently performed all measurements, and the reliability was assessed. Marginal bone loss was measured on standardized periapical radiographs using ImageJ software (National Institutes of Health, Bethesda, Maryland, United States).

Statistical analysis

Statistical analysis was performed using the IBM SPSS Statistics for Windows, Version 26 (Released 2018; IBM Corp., Armonk, New York, United States). Continuous variables were expressed as mean and standard deviation, and categorical variables were expressed as frequencies and percentages. Comparisons between groups were performed using the independent samples t-test and the chi-square test. Survival analysis was conducted using the Kaplan-Meier method, and group differences were assessed using the log-rank test. Cox proportional hazards regression analysis was performed to identify the predictors of complications. Receiver operating characteristic (ROC) curve analysis was used to determine the optimal angulation threshold. Statistical significance was set at less than 0.05.

## Results

A total of 720 implant-supported single crowns were included in the study. The cohort consisted of both screw-retained (n = 368, 51.1%) and cement-retained restorations (n = 352, 48.9%), with comparable baseline characteristics. The demographic and clinical characteristics of the study population are summarized in Table 1. The mean age at implant placement was 52.4 ± 11.6 years. There were no statistically significant differences between the groups with respect to age, sex distribution, smoking status, implant location, bone quality, or implant dimensions (all p > 0.05), indicating comparability between the groups.

**Table 1:** Baseline demographic and clinical characteristics (n = 720). Data are presented as mean ± standard deviation or n (%), SRC denotes screw-retained crown, CRC denotes cement-retained crown, t: independent samples t-test, χ^2^: chi-square test, p < 0.05 considered statistically significant.

Characteristic	Category	Total (n = 720)	SRC (n = 368)	CRC (n = 352)	Test value	p-value
Age at placement, mean ± SD	Years	52.4 ± 11.6	51.8 ± 11.2	53.1 ± 12.0	t = -1.50	0.148
Sex, n (%)	Male	396 (55.0)	198 (53.8)	198 (56.3)	χ^2^ = 0.435	0.503
	Female	324 (45.0)	170 (46.2)	154 (43.8)		
Smoking, n (%)	Never	468 (65.0)	245 (66.6)	223 (63.4)	χ^2^ = 1.02	0.371
	Former	144 (20.0)	72 (19.6)	72 (20.5)		
	Current	108 (15.0)	51 (13.9)	57 (16.2)		
Arch, n (%)	Maxilla	396 (55.0)	194 (52.7)	202 (57.4)	χ^2^ = 1.58	0.197
	Mandible	324 (45.0)	174 (47.3)	150 (42.6)		
Position, n (%)	Molar	331 (46.0)	162 (44.0)	169 (48.0)	χ^2^ = 1.28	0.284
	Premolar	245 (34.0)	128 (34.8)	117 (33.2)		
	Anterior	144 (20.0)	78 (21.2)	66 (18.8)		
Implant, mean ± SD	Diameter (mm)	4.1 ± 0.6	4.2 ± 0.6	4.0 ± 0.6	t = 4.47	0.052
	Length (mm)	11.4 ± 1.9	11.5 ± 1.8	11.3 ± 2.0	t = 1.41	0.241
Bone quality, n (%)	I-II	389 (54.0)	202 (54.9)	187 (53.1)	χ^2^ = 0.226	0.638
	III-IV	331 (46.0)	166 (45.1)	165 (46.9)		
Angulation, n (%)	0-10° (Low)	284 (39.4)	148 (40.2)	136 (38.6)	χ^2^ = 0.216	0.671
	11-20° (Moderate)	252 (35.0)	128 (34.8)	124 (35.2)		
	>20° (High)	184 (25.6)	92 (25.0)	92 (26.1)		
Mean angulation, mean ± SD	Degrees	13.8 ± 6.4	13.5 ± 6.2	14.2 ± 6.7	t = 1.45	0.138
Mean follow-up, mean ± SD	Years	5.3 ± 1.8	5.4 ± 1.7	5.2 ± 1.9	t = 1.49	0.183

**Figure 2:**
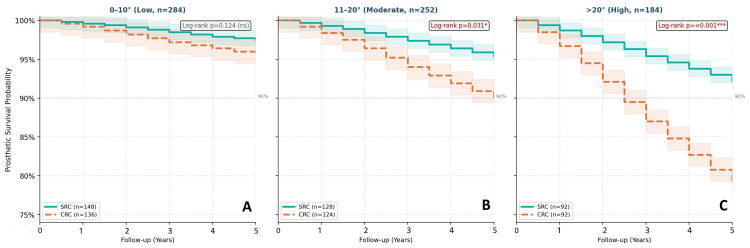
Kaplan-Meier survival curves of prosthetic survival by implant angulation and retention type. (A) 0-10°: no significant difference between screw-retained crowns (SRC) and cement-retained crowns (CRC); (B) 11-20°: SRC shows significantly higher survival than CRC; (C) >20°: CRC demonstrates significantly lower survival than SRC. Shaded areas represent confidence intervals. Survival differences were compared using the log-rank test; p < 0.05 was considered statistically significant.

Kaplan-Meier survival analysis (Figure 2) demonstrated that prosthetic survival decreased with increasing implant angulation. Screw-retained restorations showed consistently higher survival probabilities than did cement-retained restorations across all angulation categories. The difference between the groups was statistically significant (p < 0.05).

The mechanical complication rates are summarized in Table 2. At low angulation, no significant difference was observed between the groups (p = 0.363). At moderate angulation, cement-retained restorations exhibited significantly higher complication rates, particularly crown decementation (p = 0.028). At high angulation, the complication rates were markedly higher in cement-retained restorations, with statistically significant differences observed (p < 0.001).

**Table 2:** Mechanical complications by implant angulation levels in screw-retained (SRC) and cement-retained (CRC) restorations. Data are presented as n (%), comparisons performed using Fisher's exact test, * indicates statistically significant difference, and p < 0.05 is considered statistically significant. In Fisher’s exact test, no test statistic is needed as it computes probabilities directly from the hypergeometric distribution. For CRC, “screw loosening” refers to loosening of the abutment screw; for SRC, it refers to loosening of the prosthetic screw. Crown decementation and abutment fractures are reported only for CRC, as SRC does not have separate abutments.

Angulation	Group	Screw loosening, n (%)	Screw fracture, n (%)	Crown decementation, n (%)	Abutment fracture, n (%)	p-value
0-10°	SRC	4 (2.7)	1 (0.7)	0 (0)	0 (0)	0.363
	CRC	6 (4.4)	0 (0)	5 (3.7)	1 (0.7)	
11-20°	SRC	6 (4.7)	2 (1.6)	0 (0)	1 (0.8)	0.028*
	CRC	9 (7.3)	3 (2.4)	8 (6.5)	2 (1.6)	
>20°	SRC	7 (7.6)	3 (3.3)	0 (0)	2 (2.2)	<0.001*
	CRC	11 (11.9)	5 (5.4)	14 (15.2)	4 (4.3)	

Biological complication rates are shown in Table 3. No significant differences were observed at low angulation (p = 0.291). However, at moderate and high angulations, cement-retained restorations showed significantly higher rates of peri-implantitis and mucositis than screw-retained restorations (p < 0.05).

**Table 3:** Biological complications by implant angulation levels in screw-retained (SRC) and cement-retained (CRC) restorations. Data are presented as n (%), comparisons performed using Fisher's exact test, * indicates statistically significant difference, and p < 0.05 is considered statistically significant. In Fisher’s exact test, no test statistic is needed as it computes probabilities directly from the hypergeometric distribution.

Angulation	Group	Peri-implantitis, n (%)	Peri-implant mucositis, n (%)	p-value
0-10°	SRC	2 (1.4)	2 (1.4)	0.291
	CRC	4 (2.9)	3 (2.2)	
11-20°	SRC	3 (2.3)	3 (2.3)	0.041*
	CRC	7 (5.6)	6 (4.8)	
>20°	SRC	3 (2.3)	4 (4.3)	<0.001*
	CRC	9 (9.8)	8 (8.7)	

Cox proportional hazard regression analysis (Table 4) identified cement-retained restorations and high implant angulation as significant predictors of prosthetic complications. The interaction between retention type and angulation was also significant, indicating an increased risk when cement-retained restorations were used at higher angulations.

**Table 4:** Multivariable Cox proportional hazards regression analysis of factors associated with prosthetic complications. HR: hazard ratio; CI: confidence interval; CRC: cement‑retained crown; SRC: screw‑retained crown; ref: reference category Analysis was performed using Cox proportional hazards regression with a multivariable model adjusted for relevant covariates. The “unadjusted” model includes each predictor separately. The “adjusted” (interaction) model includes main effects of retention type and angulation plus their interaction term; the “fully adjusted” model additionally controls for implant position (posterior vs. anterior), arch (mandible vs. maxilla), age (per 10‑year increment), smoking status (current vs. never), bone quality (III-IV vs. I-II), and implant diameter (<4.0 mm vs. ≥4.0 mm). * p < 0.05 is considered statistically significant.

Predictor variable	HR	95% CI	p-value
Primary predictors (unadjusted)			
Retention type: CRC vs. SRC (ref)	1.42	1.14-1.77	0.002*
Angulation 11-20° vs. 0-10° (ref)	1.28	0.98-1.68	0.071
Angulation >20° vs. 0-10° (ref)	1.87	1.42-2.47	<0.001*
Interaction terms (adjusted model)			
CRC × angulation 11-20° (interaction)	1.61	1.18-2.20	0.002*
CRC × angulation >20° (interaction)	2.84	2.04-3.96	<0.001*
Covariates (fully adjusted model)			
Posterior vs. anterior position (ref)	0.91	0.72-1.15	0.381
Mandible vs. maxilla (ref)	0.95	0.74-1.22	0.621
Age (per 10-year increment)	1.08	0.96-1.22	0.208
Smoking: current vs. never (ref)	1.54	1.21-1.95	<0.001*
Bone quality III-IV vs. I-II (ref)	1.33	1.04-1.70	0.025*
Implant diameter <4.0 mm vs. ≥4.0 mm (ref)	1.19	0.88-1.62	0.245

**Figure 3:**
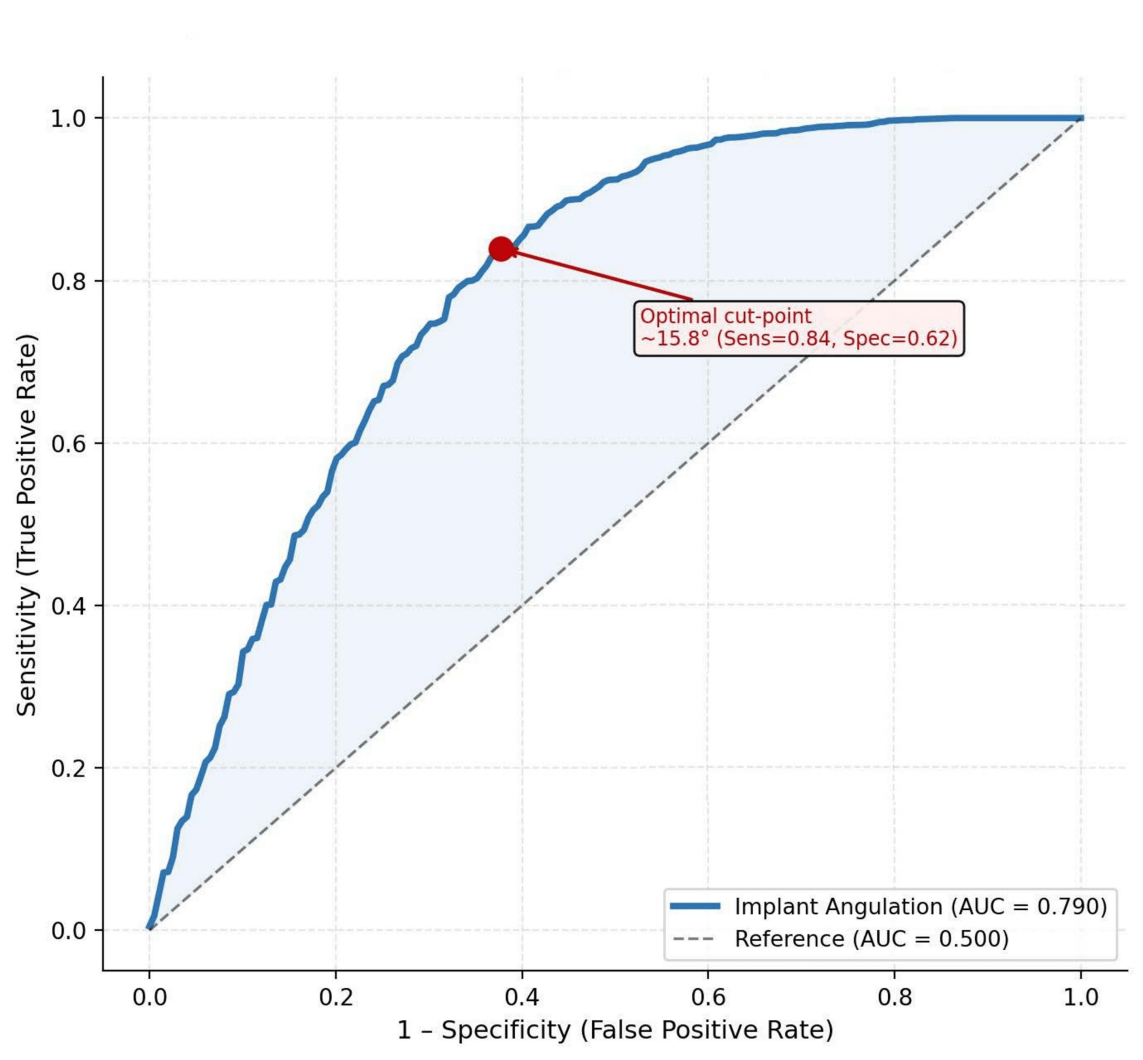
ROC curve demonstrating the diagnostic performance of implant angulation in predicting prosthetic complication risk. AUC denotes area under the curve. Receiver operating characteristic (ROC) analysis was performed to determine the optimal angulation threshold, and p < 0.05 was considered statistically significant.

ROC curve analysis (Figure 3) demonstrated the good predictive ability (area under the curve (AUC) = 0.790) of implant angulation for complication risk. A clinically relevant threshold (cut-point = -15.8°) was identified, beyond which the complication rates increased significantly.

## Discussion

The findings of our study demonstrated that both implant angulation and prosthetic retention type significantly affected the prosthetic survival and complication rates. Specifically, screw-retained restorations exhibited superior survival outcomes across all angulation categories, whereas cement-retained restorations showed a marked increase in both mechanical and biological complications with increasing angulation. The divergence between the two retention types became particularly evident beyond 20° implant angulation, suggesting the presence of a clinically relevant threshold.

The observed reduction in prosthetic survival with increasing angulation can be explained by altered biomechanical loading conditions. Excessive implant angulation leads to non-axial loading, resulting in increased stress concentration at the implant-abutment interface and surrounding bone. This predisposes restorations to mechanical complications, such as screw loosening and fracture. Previous studies have similarly reported that off-axis loading contributes to prosthetic instability and increased complication rates, particularly in cases of unfavorable implant positioning [10-12]. The present findings are consistent with these observations, demonstrating a progressive increase in complications with increasing angulation.

The superior performance of screw-retained restorations in this study may be attributed to their retrievability and the absence of cement-related complications. Cement‑retained restorations, although advantageous in terms of esthetics and passive fit, carry a risk of residual subgingival cement, which has been implicated in the literature as a major etiological factor for peri‑implant inflammation [2,13]. However, residual cement was not directly measured or quantified in the present study; therefore, the higher biological complication rates observed in cement‑retained restorations (Table 3) may be attributed to a combination of factors, including potential residual cement, impaired retrievability, and the compounding effect of implant angulation. Wilson [13] reported a strong association between excess cement and peri-implant disease, which supports the higher biological complication rates observed in the cement-retained group in the present study. Similar complications were reported by Pauletto et al. [14]. Furthermore, the inability to easily retrieve cement-retained restorations may delay diagnosis and management of complications, contributing to poorer long-term outcomes.

Mechanical complications, particularly crown decementation, were significantly higher in cement-retained restorations at moderate and high angulations [14]. This may be explained by the compromised retention due to angulation, which reduces the effective surface area for cement adhesion and increases tensile forces at the cement interface. By contrast, screw-retained restorations rely on mechanical retention through the screw joint, making them less susceptible to such failures. These findings are consistent with previous systematic evidence, which demonstrated that cement-retained implant-supported prostheses are associated with a higher incidence of both biological and technical complications when multiple variables are considered simultaneously, highlighting the influence of retention mechanisms on long-term outcomes [15].

However, previous systematic evidence has reported considerable heterogeneity in study designs and outcome reporting, making direct comparisons between screw-retained and cement-retained prostheses challenging, although both retention mechanisms have been associated with prosthodontic maintenance complications [16]. Wittneben et al. [17] reported no significant difference in survival between screw-retained and cement-retained restorations, although screw-retained designs demonstrated fewer biological and technical complications.

When quantitatively comparing our findings with recent studies that specifically address angulated implant restorations, several points of alignment and divergence emerge. Nastri et al. [18] prospectively followed patients receiving angulated screw channel (ASC) crowns versus cement‑retained crowns (mean follow‑up ~44 months) and found no implant failures in either group, with marginal bone loss low and comparable between groups. Our study similarly observed high prosthetic survival across all angulation groups, but we noted a significant increase in both mechanical and biological complications for cement‑retained restorations at angulations >20°, a threshold not specifically examined in their cohort. Shi et al. [19] reported a 100% implant survival at one‑year follow‑up and significantly lower bleeding on probing in the ASC group (11.6 ± 19.1%) compared with cemented crowns (33.3 ± 33.8%; p = 0.04), supporting our observation that screw‑retained designs may confer peri‑implant health advantages, particularly when angulation is present. In a two‑year retrospective case‑control study, Rella et al. [20] found significantly less marginal bone loss in ASC restorations (mesial: 0.45 ± 0.005 mm; distal: 0.45 ± 0.06 mm) than in cement‑retained crowns (mesial: 1.04 ± 0.27 mm; distal: 0.98 ± 0.16 mm; p < 0.01), which quantitatively corroborates our finding of higher biological complication rates (peri‑implantitis and mucositis) in the cement‑retained group at higher angulations.

Regression analysis further reinforced the role of implant angulation as an independent predictor of complications, particularly at values > 20°. The significant interaction between the retention type and angulation indicates that the negative effects of angulation are amplified in cement-retained restorations. This highlights the importance of simultaneously considering both variables during treatment planning. Similar conclusions have been drawn in predictive modeling studies, where multiple clinical variables interact to influence implant outcomes [6].

Although the Cox model adjusted for available covariates (smoking, bone quality, etc.), unmeasured confounders, including cement type, implant system heterogeneity, torque variability, and oral hygiene status, may have influenced the hazard ratios. Thus, the reported effect sizes should be interpreted with appropriate caution.

ROC analysis demonstrated that implant angulation has good predictive ability for complication risk, supporting its use as a clinical decision-making parameter. Advances in imaging and artificial intelligence have further emphasized the importance of preoperative assessment in predicting implant success. Taide et al. [7] showed that CBCT‑based machine learning models can forecast implant outcomes by integrating multiple anatomical and positional factors, including implant angulation. Although our study did not employ AI methodologies, the ROC‑derived threshold of >20° aligns well with the concept of using quantitative imaging parameters to guide prosthetic selection. Thus, the growing role of AI and predictive analytics reinforces the clinical relevance of our finding that angulation is a key predictor of complications, and it supports the routine preoperative assessment of implant position, particularly when angulation approaches or exceeds 20°, to inform the choice between screw‑retained and cement‑retained restorations.

From a biological perspective, increased angulation may also contribute to plaque accumulation and difficulties in maintaining oral hygiene, particularly in cement-retained restorations. This may explain the higher rates of peri-implantitis and mucositis observed in this study. Additionally, implant design factors and prosthetic configurations have been shown to influence peri-implant bone stability, as reported by studies [8], further supporting the multifactorial nature of the biological complications.

The clinical implications of our findings are significant. Implant angulation should be carefully considered during surgical planning, with efforts to achieve optimal positioning whenever possible. In cases where high angulation is unavoidable, screw-retained restorations are preferred because of their superior performance and lower complication rates. The use of angled screw-channel systems may further enhance the feasibility of screw-retained designs in such scenarios. In addition, meticulous cementation protocols and thorough removal of excess cement are essential when cement-retained restorations are used.

Despite its strengths, this study had certain limitations. The retrospective design introduces the possibility of selection and information bias, including potential inaccuracies or inconsistencies in electronic dental records. Variability in operator technique, implant systems (different manufacturers), and prosthetic materials (e.g., crown and cement types) may have influenced the outcomes, and these factors could not be fully standardized. Although efforts were made to control for confounding variables using multivariable regression analysis, residual confounding factors, such as patient-specific oral hygiene habits, parafunctional habits (e.g., bruxism), and differences in cementation technique, cannot be excluded. The measurement of implant angulation, while performed using calibrated examiners and CBCT, may still be subject to interobserver variability and technical limitations related to image resolution. Furthermore, the study was conducted at a single center, which may limit the generalizability of the findings to other populations with demographic or clinical characteristics. Finally, the minimum follow‑up of five years, while adequate for assessing medium‑term outcomes, may not capture very long‑term complications beyond this period. Future prospective multicenter studies with standardized protocols, blinded outcome assessment, and longer follow‑up are recommended to validate and extend these results.

## Conclusions

Within the limitations of this retrospective cohort study, implant angulation and prosthetic retention type significantly influenced the long-term outcomes of implant-supported single crowns. Greater angulation was associated with reduced prosthetic survival and increased complications. Screw-retained restorations outperformed cement-retained alternatives, particularly at higher angulations, showing fewer mechanical and biological issues. A clinically relevant threshold beyond 20 degrees was identified, above which complication risk rose markedly, especially for cement-retained crowns. These findings highlight the importance of optimal implant positioning and suggest that screw-retained restorations may be preferred when increased angulation is unavoidable, thereby aiding clinical decision-making and treatment planning. However, given the retrospective design, overgeneralization should be avoided. Future prospective multicenter studies are needed to validate these results and further refine clinical guidelines. Consistent terminology, referring to “implant-supported single crowns,” is maintained throughout.
